# Tracing long-term demographic changes: The issue of spatial scales

**DOI:** 10.1371/journal.pone.0208739

**Published:** 2019-01-02

**Authors:** Johannes Müller, Aleksandr Diachenko

**Affiliations:** 1 Institute of Pre- and Protohistoric Archaeology, Kiel University, Kiel, Germany; 2 Institute of Archaeology of the National Academy of Sciences of Ukraine, Kiev, Ukraine; University at Buffalo - The State University of New York, UNITED STATES

## Abstract

This paper deals with the analysis of long-term changes in population densities at the regional and macro-regional scale and in the density of metapopulations. The following issues concerning estimations are addressed: chronological resolution of demographic changes, estimation of the weight of values for population density in order to transform the initial values included in the sample into the values that may be compared with each other at the regional scale, calibration of the transformed values into real population densities, and the estimation of the weight of values for population density at the scales of macro-regions and for the density of metapopulations. The proposed methods are tested on demographic changes in Central Europe, Southern Scandinavia, Southeastern Europe, and the Near East. The obtained results represent major trends in demographic development, while the proposed methodology could also be applied in other wide-scale demographic analyses.

## Introduction

During the last decades, many different population estimates for prehistoric European populations were made, generally through the use of a variety of proxies. Both the local archaeological estimation of household densities on the basis of archaeological features and a prediction of prehistoric distribution patterns for absolute demographic values as well as the use of radiocarbon dates for relative palaeodemographies were employed (see [1–5] for an overview). However, the estimation of the average values for population density in macro-regions and for the density of metapopulations requires the normalization of data obtained in the related regional case studies. The presented research approaches this issue by introducing correction coefficients which normalize the data used for palaeodemographic estimations dealing with macro-regions and metapopulations.

In order to exemplify our approach, we employ data on population density estimations that were made by archaeologists in their local and regional working areas based on their expert knowledge. This data includes evaluations of depositional processes, chronology, character of data, and critical assessments of representativeness by experts of the targeted periods and areas, and therefore provides reliable values. Different population estimations were collected and used in order to describe the utility of the proposed method for the analysis of global demographic trends for parts of continental Europe and to compare them with the Near East.

## Materials and methods

**[**http://dx.doi.org/10.17504/protocols.io.vpve5n6**]**

Population density is estimated as population size divided by size of the area that population inhabits and is expressed in persons per sq. km. In our approach, literature was surveyed to obtain values of regional population density; these values were subsequently included in the analysis as they were estimated by the authors of the original studies (S1 Table). In doing so, we respect the individual approaches of the authors (see S1 Table) as representing some kind of regional expert knowledge. Within the scale of our analysis, variability that is due to the diversity of the individual methods is approximated, allowing the comparison of results, as it is recognized that the application of different methods is related to the qualities of archives in respective regions (Table 1). Further applications of the proposed methodology to significantly extended databases require critical analysis of initial palaeodemographic estimates. This will also include consideration of different estimates proposed for the same datasets. Furthermore, the increase in sample size that such an expanded study would bring decreases the estimated average values, resolving the issues of research bias and comparison of different methods of estimations, as suggested in studies of archaeological and ethnographical metapopulations [6–8].

**Table 1:** Methodologies applied for the estimations of population density.

	Methodology	Description	Examples
1	*Ecological/ethnographical estimations*	Population densities of recent non-literate societies in different ecological areas are used as proxies for similar palaeoecological areas occupied by prehistoric groups with similar subsistence techniques.	[6, 9]
2	*Ecological/ethnographical/archaeological estimations*	Carrying capacity is reconstructed with the help of ethnographic parameters and environmental reconstructions to set an upper limit of prehistoric population densities. Archaeological remains of contemporaneous sites are used (also with ethnographic parallels, e.g., of group sizes in houses) for the lower limit of absolute population densities.	[10–12]
3	*Ecological/archaeological estimations*	Archaeological information is used to reconstruct the technological level of subsistence economies of prehistoric societies. For reconstructed environments, the productivity of prehistoric groups is calculated according to their technological basis and transformed into population values and growth rates.	[13–15]
4	*Archaeological estimations based on data from domestic sites (houses*)	The number of contemporaneous households is reconstructed for “well researched” test areas, the determined number of houses per a defined spatial extent is applied to other settled regions and the household size is then estimated by ethnographic comparisons.	[16–18]
5	*Archaeological estimations based on data from domestic sites (sites)*	Reconstructed population sizes of settlements on the basis of contemporaneous houses are transferred into figures about inhabitants/hectare and this value is then applied to settlement areas detected, for example, by surveys.	[19, 20]
6	*Archaeological estimations based on data from single object types*	The number of site inhabitants is reconstructed by the processed amount of cereals, e.g., from contemporaneously used millstones, which is then transferred to likely number of individuals through calculations based on nutritional models.	[10]

The sample collected for this paper includes 42 population densities from Southeastern Europe, 33 population densities from Central Europe and Southern Scandinavia, and 56 population densities from the Near East. All these cases refer to agricultural populations, although the mentioned areas were also inhabited by hunter-gatherers. The overall size of the areas in the latter populations decreased over time, which is taken into account in estimations.

**Fig 1:**
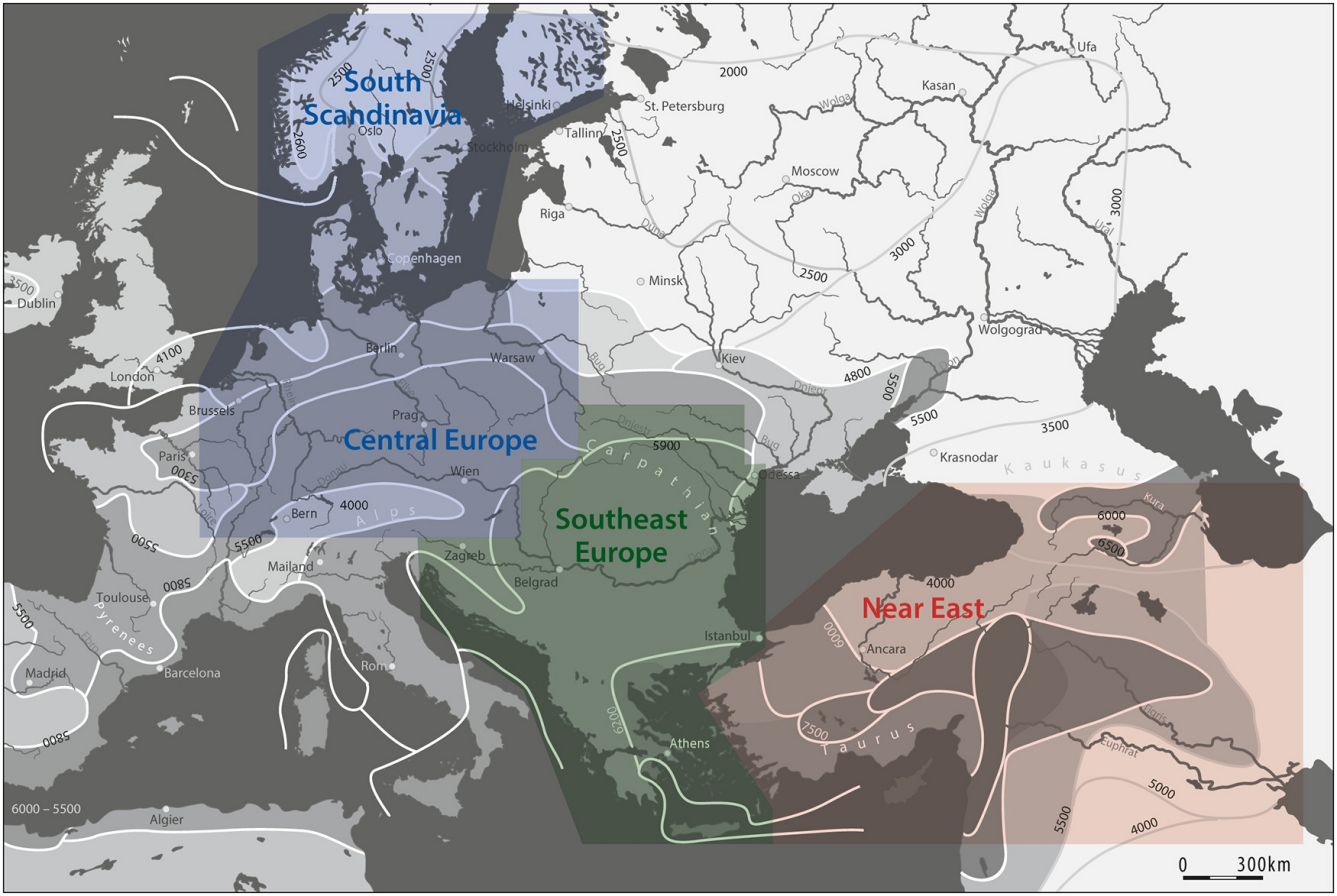
Spatial arrangement of sites in Europe and the Near East (produced using the basemap Natural Earth data by Ines Reese and Karin Winter (Graphics department of the Institute of Pre- and Protohistoric Archaeology Kiel).

Changes in population density are traced at several spatial scales. Regional density, which is also labeled ‘global’ density in the related studies [2, 3], is estimated as population density in more densely settled core areas and the surrounding un- or less settled areas, e.g. mountains. Macro-regional population density is estimated for two larger territories, i.e. Southeastern Europe and Central Europe / Southern Scandinavia (Fig 1). The density of metapopulations is represented by the values obtained for the Near East and also for Southeastern and Central Europe, and Southern Scandinavia.

The procedure of this research method considers several issues of data analysis. These can be summarized as follows:

- chronological resolution of changes;
- estimation of the weight of values for population density in order to transform the initial values included in the sample into the values that may be compared with each other at the regional scale;
- calibration of the transformed values into real population densities;
- estimation of the weight of values for population density at the scales of macro-regions and for the density of metapopulations.

The subdivision of the analyzed time span depends on the chronological resolution of the regional estimates. Considering the temporal framework for the case studies included in our sample, we decided to subdivide the overall time range into periods of 500 years. As sample size and precision of the temporal resolution of sites increase in the future, the duration of the time periods used in further developments of this demographic survey will decrease. If the chronological framework of case studies exceeds the temporal limits considered, such case studies are included into estimations of averages for two or more time periods. For instance, LBK sites in Central Europe are placed into the chronological framework of 5500 to 4900 BCE [21, 22]. Therefore, the related population density is used in estimations twice: for the time periods of 5500–5000 BCE and 5000–4500 BCE.

Case studies represent populations settling regions of different size, and for almost none of these regions were population densities estimated for the entire chronological framework of this study. Moreover, both the population size and size of the region influence the demographic averages at different spatial scales. For instance, consider a hypothetical situation in which the areas of regions A and B, which are settled by populations of different size, are estimated to make up 80% and 20% of the territory of macro-region AB respectively. The population density of the macro-region AB may then significantly deviate from the simple average value obtained as the sum of the population densities in regions A and B divided by two. Our study approaches these and other questions of economic demography by introducing correction coefficients which normalize the data used for palaeodemographic estimations dealing with macro-regions and metapopulations. With these coefficients, the following issues are questioned and addressed: To what extent does a small region that is inhabited by a large number of people, or a large area with a dispersed population contribute to the average population density at a macro-regional scale? If a certain innovation affects the increase in population density in a one region, then how does this innovation influence the population density of macro-regions and the density of metapopulations? In other words, we suggest weighting values in order to transform the initial population density data included in the sample into values that may be compared with each other at spatial scales of high orders.

All macro-regions and metapopulations considered include both hunter-gatherers and agriculturalists, groups which are characterized by significantly different densities. Hence, the impact of the related values on macro-regional densities should be separated in the analysis. Since the set of regional values included in the sample represents the density of agricultural populations of different size and the size of their regions of occupation varies, the latter variable is weighted by the coefficient ‘*p*’ which represents the proportion of the total area of the macro-region contained in that region. Multiplication of the regional population densities by this coefficient produces the ‘transformed’ values that, on the one hand, allow comparison of population densities obtained for different areas, and, on the other hand, are used in estimation of the averages for macro-regions. The average obtained for a sample of the regions that make up a macro-region at a given period of time is projected to that macro-region.

The coefficient representing the ‘weight’ of regions was estimated one time for each of regions, while the number of times a region was considered in different chronological periods is not taken into account. In other words, we propose to turn the overall occupied territory into a constant value and, hence, trace changes in population density via changes in population size. The relative sizes of the regions, as parts of the constant territory, are obtained as the result of the division of their real area by the total area of the macro-region. These contributions are then summed. Therefore, the related product of multiplication at this stage of the research does not represent the actual regional population density, but its ‘contribution’ to the macro-regional population density. The averages for agricultural populations in macro-regions are estimated as follows:

$$D_{A}\;=\;\frac{p_{1}D_{1}+p_{2}D_{2}\ldots +p_{n}D_{n}}{\sum_{i\;=\;1}^{n}p};\;p\;=\;\frac{A_{C}}{A_{M}},$$ (1)

where *D*_*A*_ is the average population density estimated for a certain time period, *p*_1_, *p*_2_, *p*_*n*_ and *D*_1_, *D*_2_, *D*_*n*_ are, respectively, coefficients transforming the values obtained for the cases in sample and densities of the cases 1, 2 and *n*. *A*_*C*_ is the size of the area in each particular case and *A*_*M*_ is the size of the macro-region.

Transformed values can be turned into real population densities with the introduction of the calibration coefficient ‘*c’*, represented by the ratio of the population density in any of the case studies to its transformed value. In the case of a sample that is as wide as possible, such as that which we plan to obtain in future investigations, the best solution for this purpose is the consideration of the most precise estimations possible in terms of original archaeological records, temporal resolution, etc. In this study, calibration coefficients for the macroregions Southeastern Europe and Central Europe and Scandinavia are equal to 1 when the initial population density values, i.e. the population densities for Thessaly from 6500–6000 BCE and for the LBK in Central Europe from 5500–5000 BCE, are considered. In the case of the Near East, the value of the calibration coefficient was estimated by random choice for the case of North Jazira in 6500 BCE in order to exemplify the application of the proposed methodology.

An estimation of the population density for a macro-region must consider the regional densities calculated for both agriculturalists and hunter-gatherers. This linearization follows the logic of estimating the macro-regional population density from several differently sized regions as discussed above. This is made possible by an introduction of the coefficient ‘*k*’ that weights the size of areas that are inhabited by populations with these subsistence strategies. The averages, summarizing the densities of agricultural populations and hunter-gatherers living in the same macro-region (*D*_*S*_), are estimated as follows.

$$D_{S}\;=\;k_{HG}D_{HG}+k_{A}D_{A};k_{HG}\;=\;\frac{A_{HG}}{A_{M}};k_{A}\;=\;\frac{A_{A}}{A_{M}},$$ (2)

where *k*_*HG*_ and *k*_*A*_ are the coefficients representing the relation of the size of an area occupied by hunter-gatherers (*A*_*HG*_) and agriculturalists (*A*_*A*_) to the total size of the macro-region.

The population density of hunter-gatherers in Europe, equal to 0.002 persons per sq. km, was borrowed from Maier et al. [7], who estimated the density of hunter-gatherers in Northern Europe in the range of 0.001–0.002 persons per 1 sq. km. These approximate values are based on the protocol, earlier proposed by Zimmermann [23], which is the closest to the procedure proposed here in terms of dealing with occupied areas and no-man’s land. Contrary, Bocquet-Appel and co-authors based their wide-scale study of Palaeolithic population dynamics in Europe on the assumption that the overall site distribution in well-researched areas is not going to change fundamentally in the future [9]. However, in areas of, for instance, high relief or extensive sediment cover, the reliability of gaps in the distribution of sites is limited [7]. Since new sites are usually discovered near known sites of the same age, the protocol of Zimmermann and co-authors, which relies on density-based delimitations of areas with intensive settlement activity, shows good results and is fairly robust with regard to the detection of new sites [23]. The population density of hunter-gatherers in the Near East, ca. 0.31 persons per sq. km, was borrowed from Hassan’s estimations [24].

Densities of metapopulations (*D*_*M*_) are estimated according to the same logic expressed in Formula (2). However, instead of densities estimated for agriculturalists and hunter-gatherers, the weighting of population densities for macro-regions (*D*_*S*1_ and *D*_*S*2_) is considered. This is expressed as follows:

$$D_{M}\;=\;k_{M1}D_{S1}+k_{M2}D_{S2};\;k_{M1}\;=\;\frac{A_{M1}}{A_{T}};k_{M2}\;=\;\frac{A_{M2}}{A_{T}},$$ (3)

where *k*_*M*1_ and *k*_*M*2_ are, respectively, coefficients reflecting the relation of macro-regions *A*_*M*1_ and *A*_*M*2_ to the total size occupied by metapopulation (*A*_*T*_).

## Results

**Fig 2:**
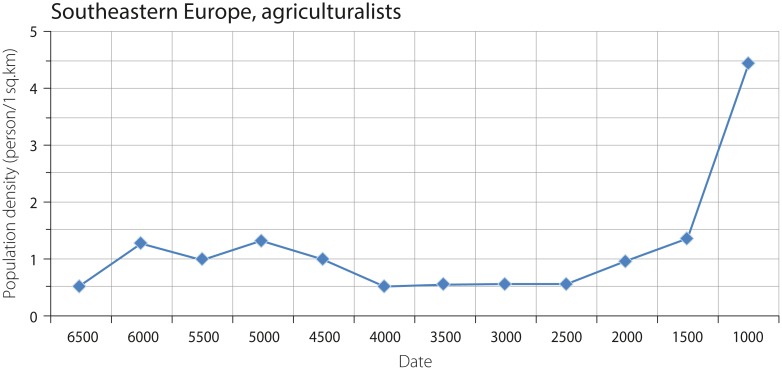
Density of agricultural populations in Southeastern Europe (the values corresponding to the dates 3500 BCE and 3000 BCE on this graph were estimated as the average for the values obtained for population densities from the ranges 4500–4000 BCE and 3000–2500 BCE, while the value corresponding to 2000 BCE is estimated as an average for population densities from the ranges 3000–2500 BCE and 2000–1500 BCE).

Let us consider the obtained results. Since the chronological resolution of our example considers time periods of 500 years, only the most significant technological and social innovations may be considered as causes of increase in population density. Fig 2 represents the changes in the population density of agriculturalists in Southeastern Europe. The graph shows two peaks. The first represents the increase in density from 0.5 persons per sq. km to ca. 1.0–1.3 persons per sq. km in the range from 6500 to 4500 BCE. The second peak shows an increase in density from ca. 1.4 to ca. 4.4 persons per q. km from 2000–1500 BCE to 1500–1000 BCE. The first peak corresponds to the spread of agriculture in Southeastern and Central Europe and the introduction of copper to the macro-region (e.g. [25]). The second peak covers the time range corresponding to the appearance of iron artefacts in Southeastern Europe. The impact of iron tools on economic productivity, however, rather corresponds to the time range of 1000–500 BCE. Hence, the observed increase in density between 1500 and 1000 BCE may be a result of a bias in our sample since it mainly includes sites in Greece.

A slight decrease in population density from 5500–5000 BCE to 5000–4500 BCE may be explained by the abandonment of tells in the latter period of time. However, it should be noted that, since the sample does not include the cases corresponding to the time periods of 4500–3500 BCE and 3500–3000 BCE, the related values for population densities were estimated as averages for preceding and subsequent time periods, i.e. 5000–4500 BCE and 3000–2500 BCE. Another (and, probably, more reliable) way to resolve the issue of ‘gaps’ resulting from a lack of regional estimates associated to certain time periods is the consideration of rate of population growth multiplied by the population density in a preceding time range [21]. Averages obtained from preceding and subsequent time periods were also estimated for the value of population density corresponding to 2000 BCE. Since the sample includes only one case for the period of time between 2500 BCE and 2000 BCE, the related value of 10 persons per sq. km (if the latter is correct—Benta, see: [26]) seems to be an overestimation in terms of its extrapolation to agricultural populations of the macro-region, i.e. the average macro-regional population density is projected from one regional value.

**Fig 3:**
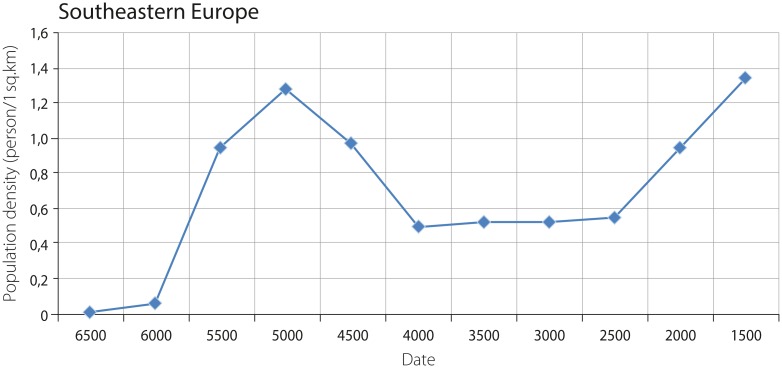
Combined density of agricultural and hunter-gatherer populations in Southeastern Europe (one should consider that the values corresponding to the dates 3500 BCE, 3000 BCE and 2000 BCE are estimated as transformed averages: See Fig 2).

Two peaks of population density, respectively 1.3 and 1.3 persons per sq. km, combining hunter-gatherer and agricultural populations in Southeastern Europe, correspond to the time periods 5500–5000 BCE and 2000–1500 BCE (Fig 3).

**Fig 4:**
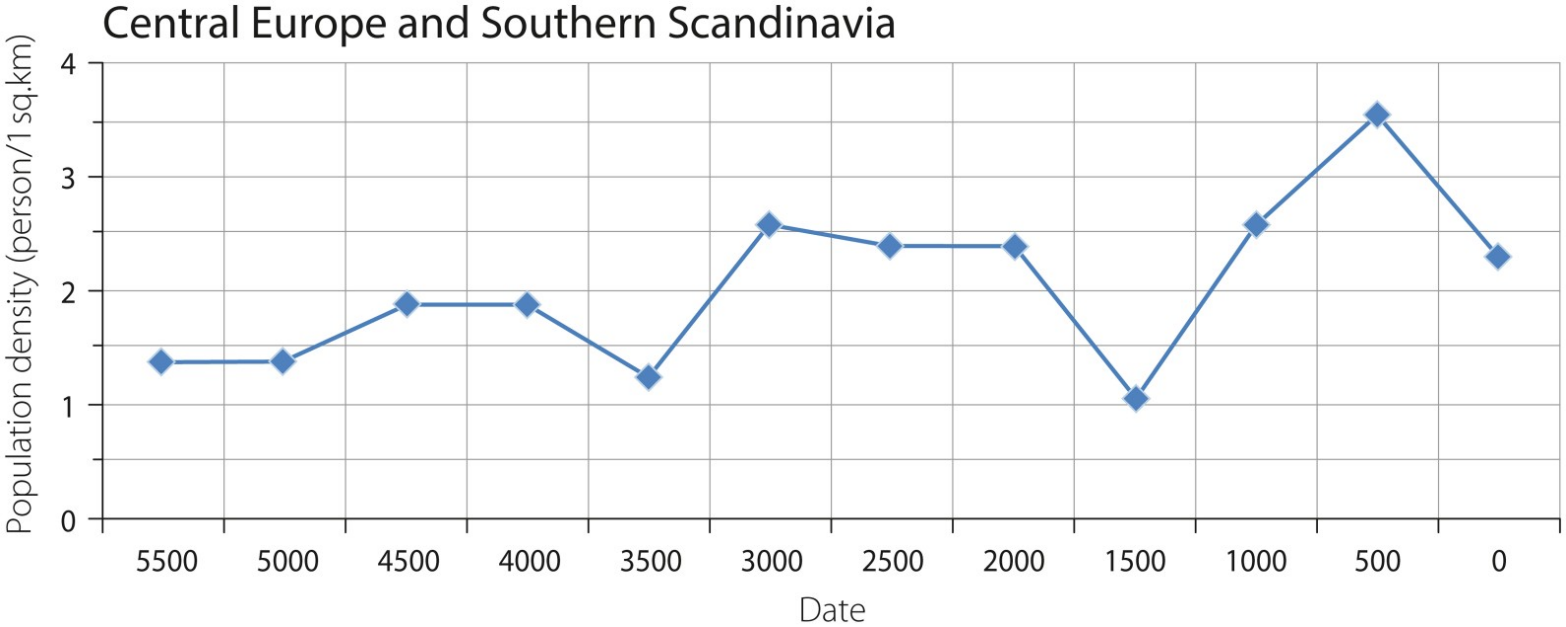
Density of agricultural populations in Central Europe and Southern Scandinavia.

An increase in the density of agricultural populations in Central Europe and Southern Scandinavia, reaching 1.9, 2.4–2.6 and 3.6 persons per sq. km, is noted for the periods in the range of 5000–4000 BCE, 3500–2000 BCE and 1000–500 BCE, respectively (Fig 4).

**Fig 5:**
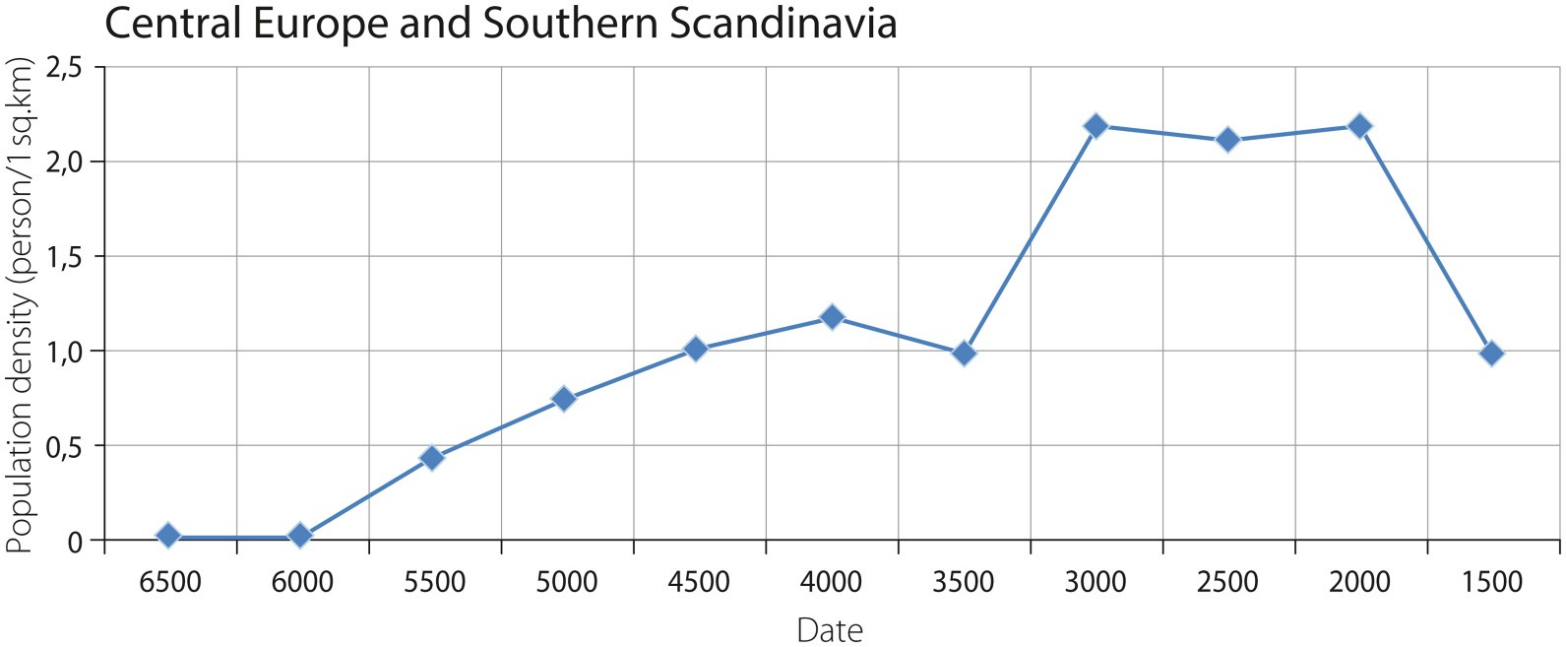
Combined density of agricultural and hunter-gatherer populations in Central Europe and Southern Scandinavia.

The macro-regional density of the combined agricultural and hunter-gatherer populations in Central Europe and Southern Scandinavia is characterized by an increase from 0.002 persons per sq. km at 6500–6000 BCE to ca. 1.2 persons per sq. km at 4000 BCE. The highest peak is estimated to 2.1–2.2 persons per 1 sq. km at the range of 3500–2000 BCE. Both increases are followed by a decrease in density to ca. 1 person per sq. km (Fig 5).

**Fig 6:**
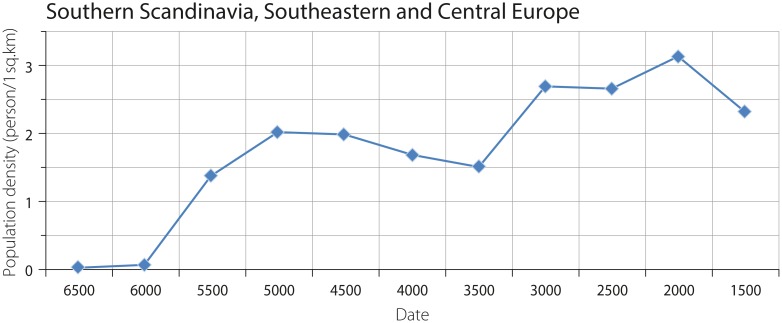
Combined density of agricultural and hunter-gatherer populations in Southern Scandinavia, Southeastern, and Central Europe (one should consider that the values corresponding to the dates 3500 BCE, 3000 BCE and 2000 BCE are estimated as transformed averages: See Fig 2).

The combined population density in Southern Scandinavia and Southeastern, and Central Europe is characterized by an S-shaped increase from ca. 0.06 persons per sq. km at 6000 BCE to ca. 2 persons per sq. km at the range of 5500–4500 BCE. The subsequent decrease is followed by a new increase in density, reaching 2.7–3.1 persons per sq. km at 3500–2000 BCE (Fig 6).

**Fig 7:**
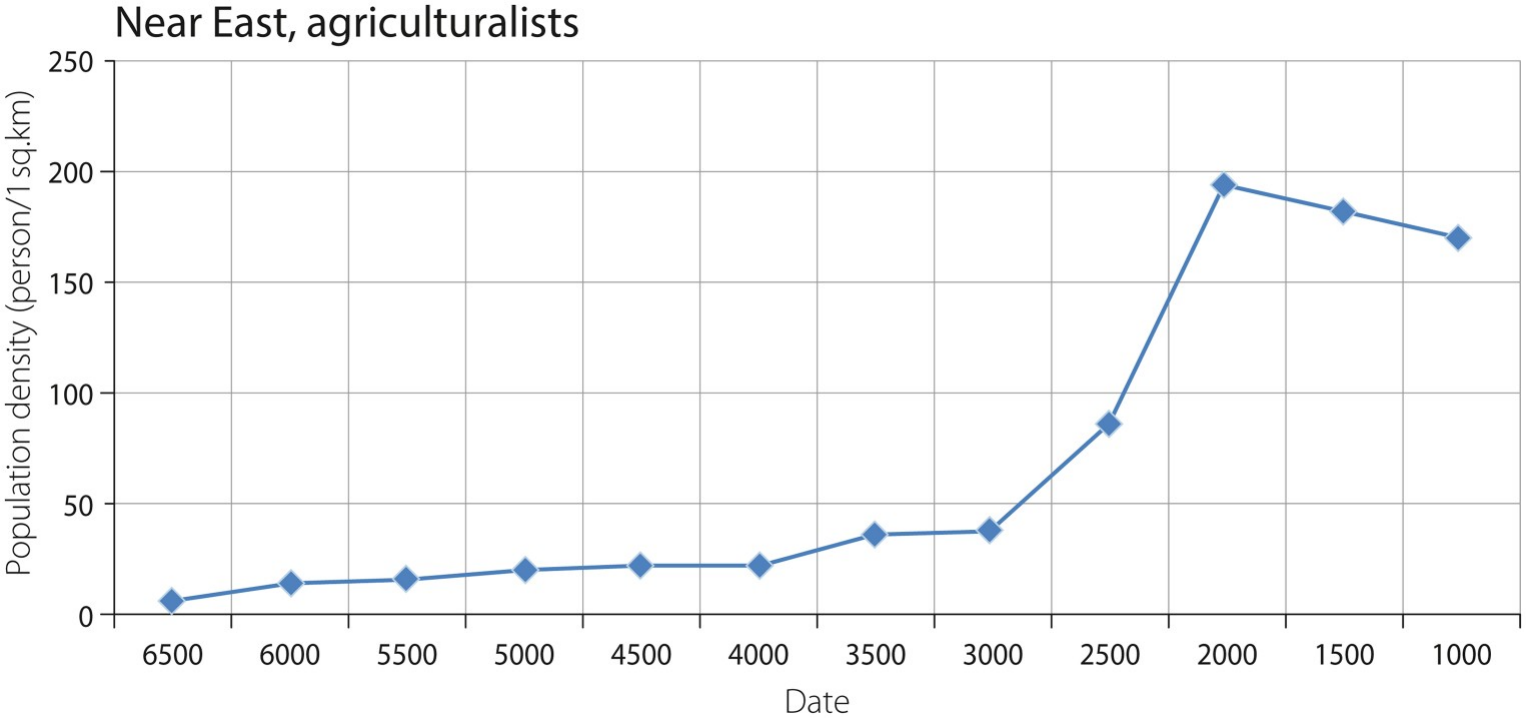
Density of agricultural populations in the Near East.

**Fig 8:**
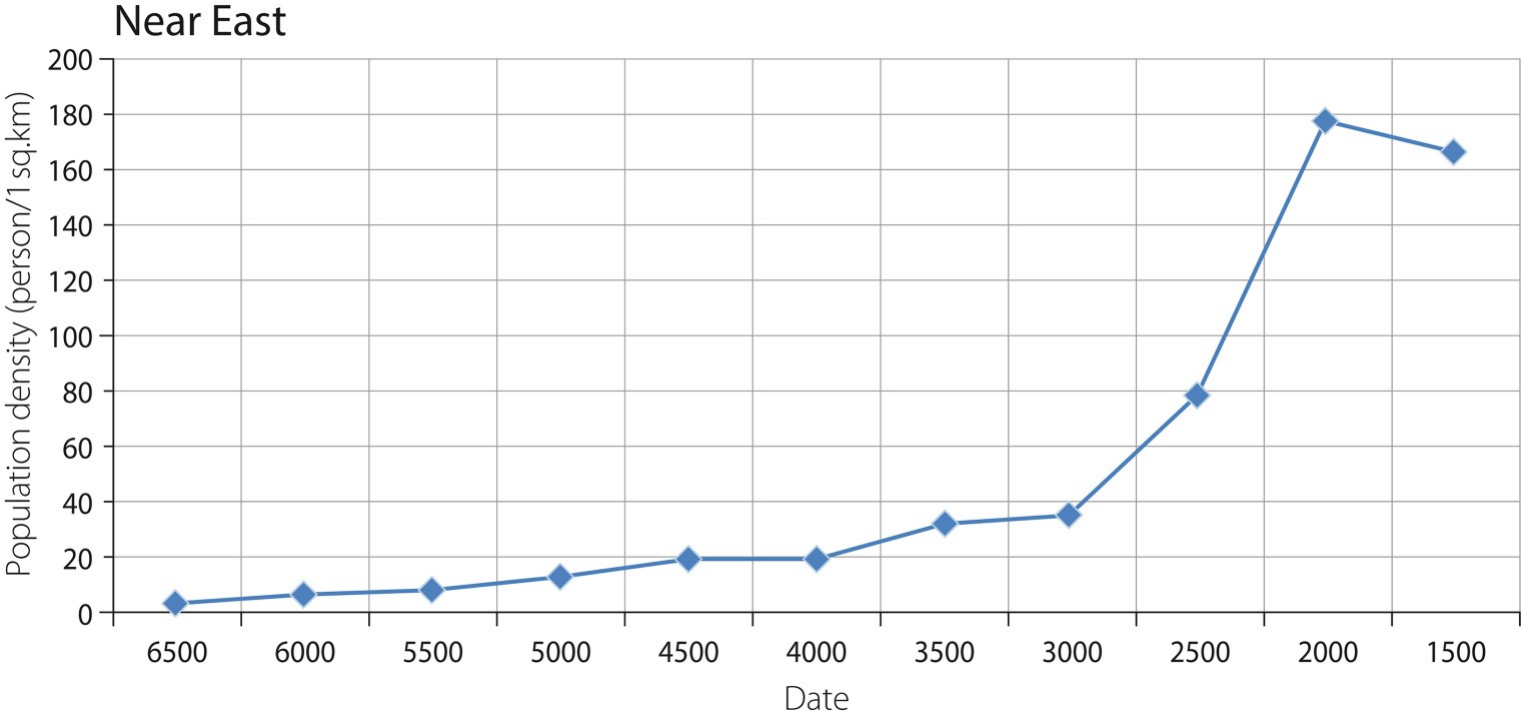
Combined density of agricultural and hunter-gatherer populations in the Near East.

The graphs showing the trajectories of change in population density of agriculturalists and combined agriculturalists and hunter-gatherers in the Near East are characterized by almost identical shapes (with the difference of a more rapid increase in combined density around 5000–4500 BCE), but they are different in values (Figs 7 and 8). The values obtained for agriculturalists and agriculturalists and hunter-gatherers combined increase from ca. 5.5 and ca. 1.7 persons per sq. km in 6500 BCE to 37.5 and 34.4 persons per sq. km in 3000 BCE, respectively. This relatively slight increase, as shown by the graph, is followed by intensive growth, reaching a peak of ca. 193.9 and ca. 177.8 persons per sq. km in 2500–2000 BCE. The subsequent decrease in population density is characterized by values of 182.1 and ca. 167 persons per sq. km from 2000–1500 BCE.

## Discussion

The population estimates presented describe the demographic dimension of the Southeast European development in the first generations after the introduction of agriculture and subsequent copper metallurgy. The densities are comparable to later Bronze Age developments around 1500 BCE. Furthermore, the decline in population happened much later then estimated by Boquet-Appel [27], which brings their model of the adaptation and disintegration structure of demography after neolithisation processes into question. Instead, the social dimension of these societies and their internal development seem to influence the demographic values more than ecodeterministic adaptation processes [28].

A similar picture arises for Central Europe and Southern Scandinavia (Fig 5). The continuous population increase was not followed by a steep reduction in population densities. Furthermore, a main increase in population is detected in the late fourth and the early third millennium BCE, which is also not linked to ecological aspects. Again, the introduction of new technologies (such as wheel and wagon, ploughing techniques) influenced the population increase. On a broader European scale, such a “system change” around 3500 BCE also seems to be obvious when taking the Southeast European, Central European and Scandinavian estimations together. On the continental scale of Europe, in our opinion the two main developments of European prehistory are thus underlined: the early agricultural and metallurgical system (ca. 6000–3500 BCE) and the late Chalcolithic/Early Bronze Age system (3500–1500 BCE). In addition to this difference, a comparison with the Near Eastern development reveals that populations are 5–10 times more dense in the Near East at ca. 3000 BCE, and that the subsequent major population increase is linked to urbanization processes that are not known from prehistoric Europe.

Further work on estimations of population density at different spatial scales will consider significant elaborations of the database. This includes consideration and critical analysis of the huge amount of regional data which is available from numerous regional case studies conducted in the last decades but was obtained under different methodological frameworks. The enlargement of the database will significantly increase the chronological resolution of the related studies and, hence, allow more precise analysis of the palaeodemographic trends. These may then be compared with the results of large-scale analyses based on independent records, e.g., distributions of the radiocarbon dates or DNA evidence (e.g. [5, 16, 29–37]).

Normalization of the initial population estimates, which is suggested in this paper, allows the analysis of positive and negative deviations from the macro-regional averages. This leads to proper identification of more settled and less settled areas, allowing the consideration of migratory behavior, centers and peripheries defined by demographic properties of the inhabiting populations, and current diffusion models. The latter task is a crucial point in the consideration of demographic development in the peripheral regions as is exemplified, for instance, by the recent demographic estimates obtained for Tripolye mega-sites in modern Ukraine and Moldova, where population density in the frontier of agricultural Southeastern Europe was found to exceed the related values obtained for the core areas (the related estimations are presented and discussed in [38, 39]).

## Supporting information

S1 Table Values of regional population density. These values were included in the analysis as they were estimated by the authors of the original studies.
(XLSX)
